# Potential Antigens Involved in Delayed Xenograft Rejection in a Ggta1/Cmah Dko Pig-to-Monkey Model

**DOI:** 10.1038/s41598-017-10805-0

**Published:** 2017-08-30

**Authors:** Junfang Zhang, Chongwei Xie, Ying Lu, Ming Zhou, Zepeng Qu, Da Yao, Chuanghua Qiu, Jia Xu, Dengke Pan, Yifan Dai, Hidetaka Hara, David K. C. Cooper, Shanshan Ma, Mingtao Li, Zhiming Cai, Lisha Mou

**Affiliations:** 1 Shenzhen Xenotransplantation Medical Engineering Research and Development Center, Institute of Translational Medicine, Shenzhen Second People’s Hospital, School of medicine, Shenzhen University, Shenzhen, Guangdong, 518060 China; 2 Department of Biochemistry, Zhongshan School of Medicine, Sun Yat-sen University, Guangzhou, 510080 China; 3 Key Laboratory of Farm Animal Genetic Resource and Germplasm Innovation of Ministry of Agriculture, Institute of Animal Science, Chinese Academy of Agricultural Sciences, Beijing, 100193 China; 4 Jiangsu Key Laboratory of Xenotransplantation, Nanjing Medical University, Nanjing, 210029 China; 5 Xenotransplantation Program/Department of Surgery, The University of Alabama at Birmingham, Birmingham, AL 35233 USA

## Abstract

When hyperacute rejection is avoided by deletion of Gal expression in the pig, delayed xenograft rejection (DXR) becomes a major immunologic barrier to successful xenotransplantation. This study was to investigate the potential antigens involved in DXR. We isolated primary renal microvascular endothelial cells (RMEC) and aortic endothelial cells (AEC) from a GGTA1/CMAH double-knockout (DKO) pig (and a GGTA1-KO pig) and immunized cynomolgus monkeys with both of these cells. After sensitization, monkey serum antibody binding and cytotoxicity to RMEC was significantly higher than to AEC(p < 0.05), suggesting that RMEC are more immunogenic than AEC. Transcriptome sequencing of GGTA1/CMAH DKO pigs indicated that the expression of 1,500 genes was higher in RMEC than in AEC, while expression of 896 genes was lower. Next, we selected 101 candidate genes expressed only in pig RMEC, but not in pig AEC or in monkey or human RMEC. When these genes were knocked out individually in GGTA1/CMAH DKO RMEC, 32 genes were associated with reduced antibody binding, indicating that these genes might be primary immunologic targets involved in DXR. These genes may be important candidates for deletion in producing pigs against which there is a reduced primate immune response in pig kidney xenograft.

## Introduction

Kidney transplantation is the current optimal therapy for end-stage renal disease, but many patients do not have the opportunity of obtaining a suitable donor kidney due to a critical shortage of deceased human organs^1, 2^. Genetically-modified pigs could be an alternative source of organs^3, 4^. There have been recent encouraging results following life-supporting genetically-engineered pig kidney transplantation in nonhuman primates (NHPs), with survival now extending for several months^5–7^.The factors contributing to these improving results include the genetically-engineering of pigs, a costimulation blockade-based immunosuppressive regimen, and anti-inflammatory therapy. However, delayed xenograft rejection (DXR) and the development of a thrombotic microangiopathy in the graft (characterized by fibrin-platelet thrombi in the microvasculature causing ischemic injury in the graft) have been observed in both cardiac and renal xenografts and remain problematic^8^.

The microvascular circulation comprises vessels that are <150 μm and includes arterioles, capillaries, and venules^9, 10^, The microcirculation provides nutrition and oxygen to tissues and maintains hydrostatic pressure, which is essential for normal tissue function^11^. Some clinical studies have shown that loss of the microvascular circulation precedes (and may predispose allografts to) chronic rejection and/or graft failure^12, 13^. These studies suggest that a functional microvascular system is essential for the health of a solid organ transplant. Preservation of an intact microcirculation may represent a novel therapeutic strategy to prevent or attenuate chronic rejection^14^.

However, the endothelial lining of the vasculature of the graft is a major target for the host’s immune response, characterized by antibody-mediated rejection and/or thrombotic microangiopathy^15, 16^. Preformed and induced antibody directed toward the vascular endothelium is considered to be the primary immune mechanism in the development of DXR, which is believed to result from chronic activation or injury to the vascular endothelium mediated by antibody binding and/or complement activation^17^. These processes promote the formation of a thrombogenic vasculature, which, if unchecked, leads to microvascular thrombosis and ischemic injury^18^.

Chronic *allograft* vasculopathy in larger vessels has long been recognized as a major limitation for the long-term survival of patients after organ transplantation^14^. However, how microvascular injury and the accompanying pathologic remodeling affects chronic rejection and graft survival is not well-understood^19, 20^. In xenotransplantation, identifying new target antigens may be important for developing new genetically-engineered pigs whose organs are resistant to chronic antibody-mediated activation of the vascular endothelium and for establishing antigen-specific tolerance^21, 22^.

We generated double-knockout (DKO) pigs deficient in expression of galactose-α1,3-galactose (Gal) (α1,3-galactosyltransferase gene knockout [GGTA1-KO] pigs) and N-glycolylneuraminic acid (Neu5Gc) (cytidine monophosphate-N-acetylneuraminic acid hydroxylase gene-knockout [CMAH-KO] pigs), thus reducing the extent of human antibody binding and antibody-mediated complement-dependent cytotoxicity significantly^23^. We also generated single-knockout pigs (GGTA1-KO). However, antibody binding to nonGal endothelial cell membrane antigens may still result in DXR and/or chronic rejection and graft loss. Identification of these porcine nonGal/nonNeu5Gc gene products may create new opportunities for genetic modification of the source pig and prevention of antibody-mediated injury to the graft.

The primary aim of the study was to immunize monkeys to pig antigens and determine whether antibodies developed to new pig antigens that had not been previously identified.

## Materials and Methods

### Animal care

All the animal experiments were approved by the Institutional Review Board on Bioethics and Biosafety of Beijing Genomics Institute (BGI-IRB) (following IACUC-approved protocols published by the Yerkes Primate Center, Atlanta, GA, USA).

All surgical procedures were performed under full inhalational anesthesia, and all efforts were made to minimize animal suffering. All of the animals were handled according to the Ministry of Health guidelines for the care and use of laboratory animals (GB 14925–2001), and all of the procedures were approved by the Laboratory Animal Ethics Committee of the Sun Yat-sen University.

### Isolation of primary pig renal microvascular endothelial cells (RMEC) and aortic endothelial cells (AEC)

We recently produced GGTA1-KO and GGTA1/CMAH DKO pigs^23^. Porcine kidneys from wild-type (WT), GGTA1-KO, and GGTA1/CMAH DKO pigs were flushed with 0.025% of collagenase type IV from Clostridium histolyticum (Sigma, St. Louis, MO) at 37 °C. Primary renal cell were isolated and cultured in Endothelial Cell Medium (Lonza, Basel, Switzerland). On day 3 post-isolation, cell sorting for CD31-positive (AbD SeroTec, Raleigh, NC) RMEC and AEC was performed using a flow cytometry cell sorter (BD Biosciences, cat.no.FACS Aria II, San Jose, CA). These cells were used within 3 to 5 passages.

### Flow cytometry

The cells were harvested and stained, as described previously^23^. All chemicals, except monoclonal antibodies, were purchased from Sigma Chemical Co. (St. Louis, MO). The following antibodies were used: anti-pig CD31 (AbD SeroTec,Kidlington, UK), donkey anti-chicken DyLight 649 antibody (Jackson ImmunoResearch, West Grove, PA), von Willebrand (AbD SeroTec),anti-pig swine leukocyte antigen (SLA) class II DR and DQ and SLA class I (AbD SeroTec). A chicken anti-Neu5Gc antibody kit (Biolegend, San Diego, CA) was used according to the manufacturer’s instructions. Briefly, for Gal staining, cells were stained with FITC-conjugated BS-IB4 lectin for 30 min at 4 °C; unstained cells were used as a negative control. Neu5Gc was stained with an anti-Neu5Gc antibody kit (Biolegend), with secondary and tertiary antibodies (Jackson ImmunoResearch), following the manufacturer’s protocols. Flow cytometric data were collected using BD FACS AriaI and CFlow software (Accuri, Ann Arbor, MI).

### Immunization of cynomolgus monkeys

Three female cynomolgus macaques (*Macaca fascicularis*, 3–4-years-old, 4 kg) were obtained from Landau Biotech (Guangdong, China), and maintained in the South China Primate R&D Center (Guangzhou, China). Immunization was performed by subcutaneous injection in the axilla using 2 × 10^7^ RMEC and 2 × 10^7^AEC, respectively, mixed in PBS to a total volume of 1 mL. Each monkey received injections of both RMEC (one axilla) and AEC (alternate axilla) from DKO pigs. Three injections were administered to each monkey at 0, 3, and 7 weeks. (We reasoned that exposure to both RMEC and AEC represented the EC exposure to a pig kidney graft). Three further monkey received identical treatment, but with cells from a GGTA1-KO pig.

### Antibody binding to RMEC and AEC

Blood samples were obtained from the immunized monkeys, and serum was heat-inactivated at 56 °C for 30 min. A 10% mixture of serum was incubated with 2 × 10^5^ pig cells for 30 min at 4 °C, washed × 3 with PBS, and stained with FITC-conjugated goat anti-human IgG or IgM (Invitrogen, Carlsbad, CA). Cells were washed again and analyzed by flow cytometry. The extent of IgG or IgM binding was evaluated by relative mean fluorescence intensity (MFI), using the following formula:-

$$\text{Relative MFI}\,=\,(\text{actual MFI})/(\text{MFI obtained with secondary antibodies without serum})$$

### Complement-dependent cytotoxicity (CDC) assay

RMEC and AEC (2 × 10^6^) were incubated with 10% serum samples in 200 µl for 2 h at 37 °C. After washing twice with PBS, the cells were incubated with propidium iodide (PI, 1:1000; Invitrogen) for 15 min at 4 °C. After washing, the treated cells were analyzed by flow cytometry. PI-positive cells indicated cell death. The percentage cytotoxicity was calculated by the following formula:

$$ \% {\rm{cytotoxicity}}\,=\,({\rm{A}}-{\rm{B}})/(100-{\rm{B}})\,\times 100 \% $$

where A represents the percentage of PI-positive cells incubated with serum, and B represents the percentage of PI-positive cells incubated with heat-inactivated serum.

### Transcriptome sequencing

Six kinds of cells were used for transcriptome sequencing, (i) GGTA1-KO pig AEC and RMEC, (ii) GGTA1/CMAH DKO pig AEC and RMEC, (iii) cynomolgus monkey RMEC (we isolated and cultured in Endothelial Cell Medium), and (iv) human RMEC (HRGEC, bought from ScienCell Research Laboratories, Carlsbad, CA; cultured in Endothelial Cell Medium). Total RNA was extracted using TRIzol solution (Invitrogen, Waltham, MA USA), according to the manufacturer’s protocol. RNA sequencing libraries were constructed using TruSeq RNA Sample Preparation Kit V2 (Illumina, San Diego, CA, USA), following the manufacturer’s protocol. In brief, RNA concentration was measured by Nanodrop and the quality was measured by agarose and Agilent 2100. The RNA sample passed quality control before library preparation. Following purification, the mRNA was fragmented into small pieces using divalent cations at 94 °C for 5 min. The cleaved RNA fragments were copied into first strand cDNA using reverse transcriptase and random primers. This was followed by second strand cDNA synthesis using DNA polymerase I and RNase H. (These cDNA fragments undergo an end-repair process, the addition of a single ‘A’ base, and then ligation of the adapters.) The products were then purified and enriched by PCR (15-cycle) to create the final cDNA library. After purification, quantification, and validation, validated DNA libraries were sequenced on an Illumina Sequencing System (HiSeq. 2000, San Diego, CA, USA), following the manufacturer’s standard workflow (Vazyme Biotech, Nanjing, China).

### Small guide RNA (sgRNA) design and vector construction

sgRNA targeting candidate genes were designed following protocols described previously^18^. The cas9-coding DNA fragment was synthesized and cloned into the pMD-18T vector (Takara, Dalian, Liaoning, China). A cytomegalovirus (CMV) promoter was used to drive transcription of Cas9 in the vector. The U6-sgRNA fragment was synthesized and cloned into the pMD-18T vector. Two BsaI restriction sites were introduced into the region between the U6 promoter and sgRNA tail. For sgRNA vector construction, two complementary oligo DNAs were synthesized and then annealed to a double-strand DNA, and ligated to the BsaI site of the U6-sgRNA vector to form an integral sgRNA-expressing frame.

### Statistical analysis

GraphPad Prism 5 (GraphPad Software, La Jolla, CA, USA) was used for data analysis. Data are presented as the mean ± SEM. A two-tailed Student’s t test was used for analysis of the differences between the groups. A p value of <0.05 was considered statistically significant.

## Results

### Features of RMEC and AEC from GGTA1/CMAH DKO pigs

**Figure 1:**
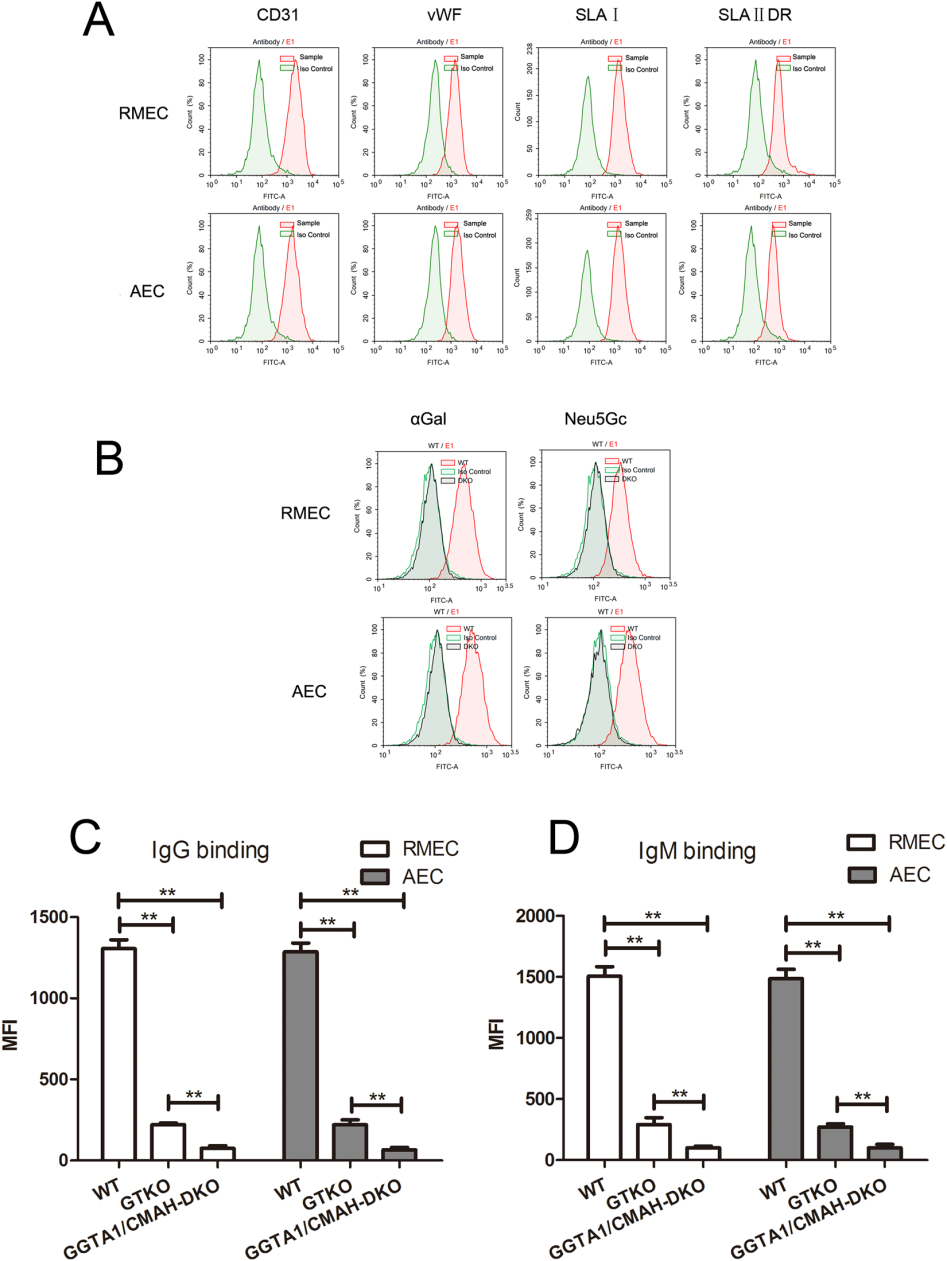
Identification of primary RMEC and AEC from GGTA1/CMAH DKO pigs (**A**) Expression of CD31, von Willebrand factor(vWF), SLA class I (SLAI), and SLA class II DR (SLA II DR) on DKO RMEC and AEC was confirmed by flow cytometry. (the ‘controls’are isotype controls) (**B**) Expression of Gal and Neu5Gc on WT and DKO RMEC and AEC was confirmed by staining with BS-IB4 lectin and an anti-Neu5Gc monoclonal antibody, respectively. (**C**) The human serum IgG binding to the WT, GGTA1-KO, and DKO pigs RMEC and AEC respectively. (**D**) The human serum IgM binding to the WT, GGTA1-KO, and DKO pigs RMEC and AEC respectively.

Recently, we obtained 11 live bi-allelic GGTA1/CMAH DKO piglets with identical phenotype by CRISPR/Cas9^16^. RMEC and AEC were isolated from the renal microvascular endothelium and aortic endothelium, respectively. Cell sorting for CD31-positive RMEC and AEC was performed using flow cytometry cell sorting, and expression of von Willebrand factor (vWF), SLA class I, and SLA class II DR were confirmed as markers of vascular endothelial cell^24^ (Fig. 1A). The REMC and AEC which we used were both primary cells, and they expressed MHC class II antigens. The cells were used under static conditions, not after stimulation. Expression of SLA class II in primary cells was greater than after passage (Supplementary Fig. F). DKO RMEC and AEC were deficient in both Gal and Neu5Gc compared with WT pig cells (Fig. 1B). We determined the human serum antibody binding to WT, GGTA1-KO, and DKO pig RMEC and AEC, respectively. Both IgG and IgM binding to DKO pig cells were significantly lower than to GGTA1-KO pig cells (Fig. 1C,D).

It has been reported that there is greater antibody binding to CMAH-KO pig cells in baboons, presumably because the absence of Neu5Gc expression may have exposed neoantigens to which monkey serum binds. However, testing of monkey sera to GGTA1-KO and GGTA1-KO/CMAH-KO (DKO) pig cells demonstrated no significant difference in binding to the two cell types (Supplementary Fig. A,B).

### Immunogenicity test by sensitizing monkeys with DKO RMEC and AEC

**Figure 2:**
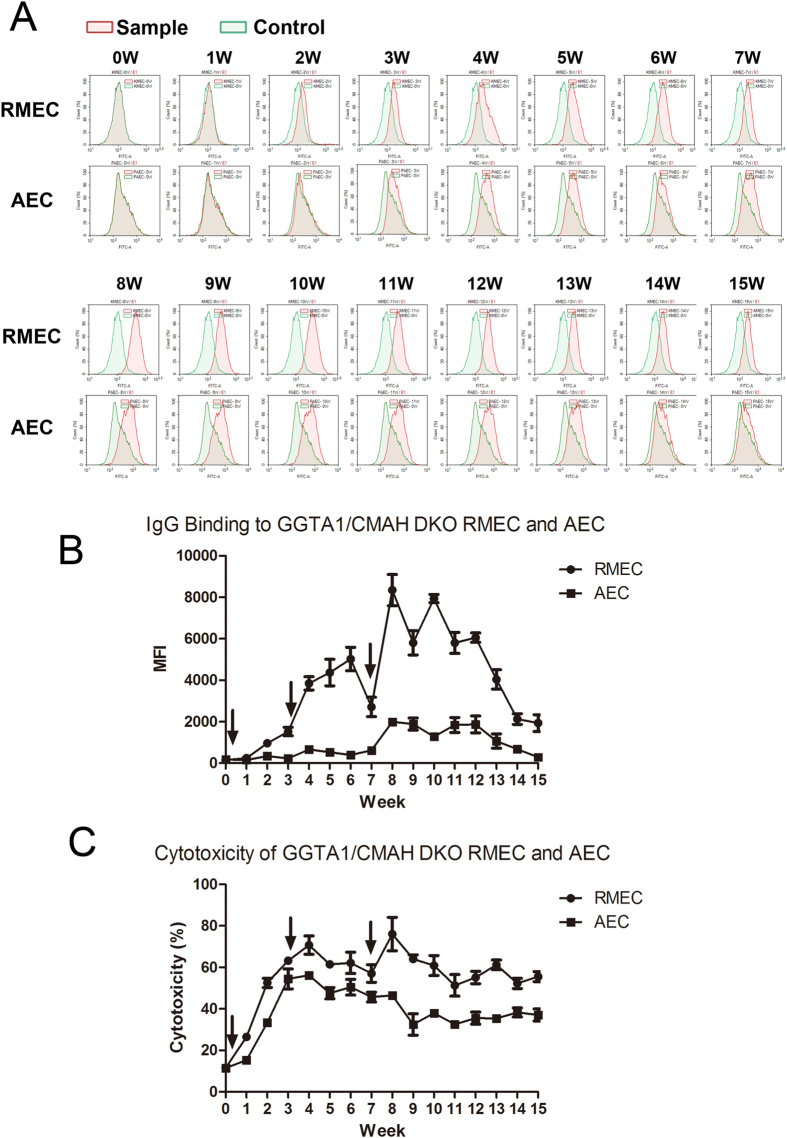
Monkey serum antibody binding to, and CDC of, DKO RMEC and AEC (**A**) Sera were collected every week for 15 weeks for IgG binding to RMEC and AEC measured by flow cytometry. Histograms in green (control) indicate antibody binding before immunization (0 week), histograms in red indicate antibody binding of immunized monkey serum at the time indicated. (**B**) Antibody binding to DKO RMEC and AEC using the same immunized serum. Binding to RMEC was significantly higher than to AEC (p < 0.05).(**C**) Percentage cell death (% cytotoxicity) of DKO RMEC and AEC. Cell death of RMEC was significantly greater than of AEC (p < 0.05).

Three female cynomolgus monkeys were sensitized by subcutaneous injection in the axilla using 2 × 10^7^ DKO RMEC and 2 × 10^7^ DKO AEC. (We reasoned that exposure to both RMEC and AEC represented the EC exposure to a pig kidney graft). Three injections were administered to each monkey at 0, 3, and 7 weeks. Serum samples were collected every week for the antibody binding assay (Fig. 2A). Statistical analysis indicated that IgG binding to RMEC was significantly greater than to AEC after immunization (p < 0.05) (Fig. 2B), suggesting a greater immunogenicity of RMEC. Furthermore, death of RMEC, determined by the CDC assay, was also significantly greater than of AEC (p < 0.05) (Fig. 2C).

We also sensitized a monkey with GGTA1-KO RMEC and AEC. Serum was collected for IgG binding and cytotoxicity. After immunization, IgG binding to GGTA1-KO RMEC was significantly greater than to GGTA1-KO AEC (p < 0.05) (Supplementary Fig. C,D). Death of GGTA1-KO RMEC, determined by the CDC assay, was also significantly greater than of GGTA1-KO AEC (p < 0.05) (Supplementary Fig. E). These results suggested a greater immunogenicity of RMEC than of AEC, which correlated with the data using DKO cells.

### Differences in gene expression between DKO RMEC and AEC

**Figure 3:**
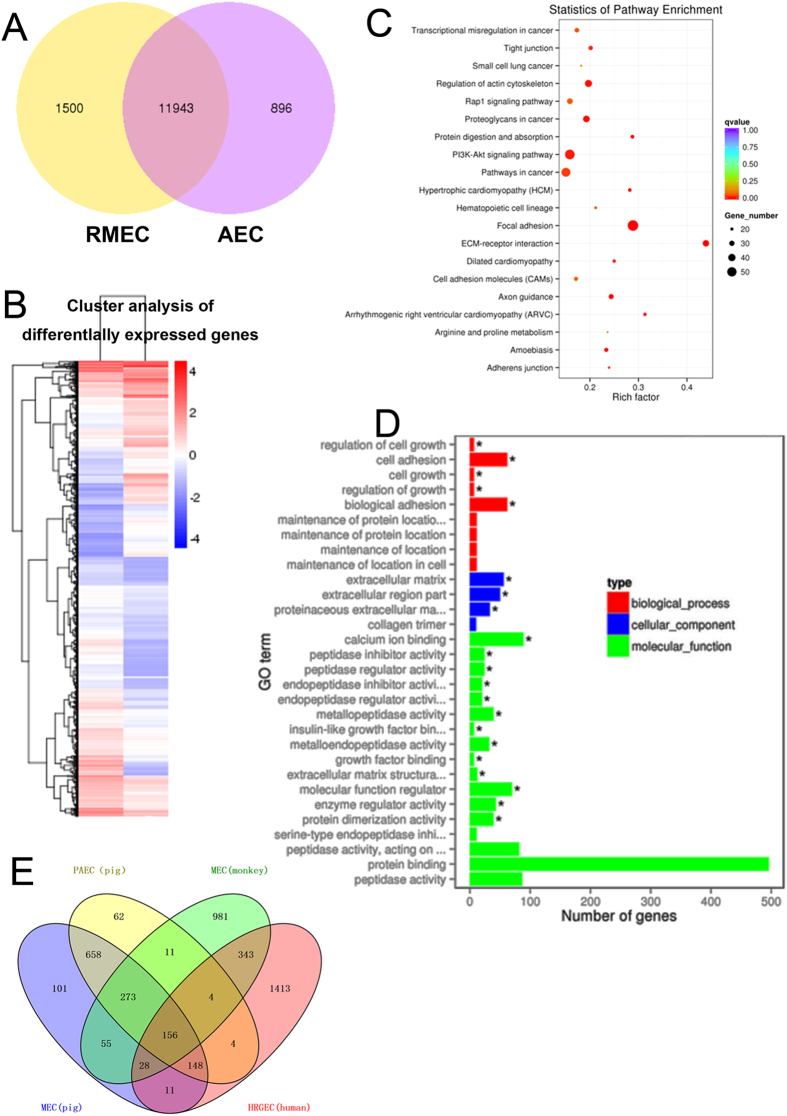
Differences in gene expression between DKO RMEC and AEC (**A**) Transcriptome sequencing was carried out to compare differences in gene expression between GGTA1/CMAH DKO RMEC and AEC, As illustrated in the Venn diagram, 1,500 genes were more highly expressed in RMEC, while 896 genes were more highly expressed in AEC. (**B**) Cluster analysis of the differentially-expressed genes in RMEC and AEC. (**C**) Gene ontology included molecular function, biological process, and cellular components of the differentially-expressed genes. (**D**) KEGG (Kyoto Encyclopedia of Genes and Genomes) analysis of the most important biochemical metabolic pathways and signal transduction pathways of differentially-expressed genes. The asterisk indicates a significant difference (**E**) The 101 genes were expressed only in pig RMEC, but not in pig AEC or cynomolgus monkey or human RMEC. These genes are both tissue-specific and species-specific.

Although both RMEC and AEC are vascular endothelial cells, they are not identical, and so we carried out transcriptome sequencing to compare gene expression. In RMEC, 1,500 genes were expressed at a higher level than in AEC, while 896 genes were expressed at a lower level (Fig. 3A). These differentially-expressed genes (between RMEC and AEC) were analyzed by clustering to show their relative incidence (Fig. 3B). Two systems were used to investigate these differentially-expressed genes. (i) Gene Ontology included molecular function, biological process, and cellular components of the differentially-expressed genes. (ii) KEGG (Kyoto Encyclopedia of Genes and Genomes) analysis included the most important biochemical metabolic pathways and signal transduction pathways of the differentially-expressed genes. The differentially-expressed genes were mainly related to cell adhesion and biological adhesion (Fig. 3C), especially in the extracellular matrix-receptor interaction and focal adhesion pathways (Fig. 3D).

Human serum antibody binding to DKO pig cells was significantly lower than to GGTA1-KO pig cells (Fig. 1C,D). Neu5Gc will have to be knocked-out from the pig genome when organs are transplanted into humans. Pig-to-NHP transplantation is the most suitable experimental model before clinical trials are undertaken. As monkeys express Neu5Gc, knockout of Neu5Gc in the donor pig may expose new antigens against which monkeys have antibodies. To investigate whether new antigens may be exposed when Neu5Gc is knocked-out, we compared monkey serum antibody binding to DKO cells and GGTA1-KO cells. The difference was not significant (Supplementary Fig. A,B).

We also compared the transcriptome between GGTA1-KO cells and DKO cells. There were 37 genes that were expressed only in DKO RMEC, but not in GGTA1-KO RMEC (p < 0.05). This suggested that deletion of Neu5Gc did, in fact, expose some new antigens that could be bound by serum antibodies. However, none of these genes was among the candidate genes we subsequently knocked out (Supplementary Table 2).

We selected only those genes expressed in pig RMEC, but not in pig AEC, as these genes may be associated with the immunogenic differences documented between these two cell types. We also compared the expression levels of these RMEC genes in three different species (pig, monkey, and human). Genes expressed only in pig RMEC, but not in monkey or human RMEC, may be important immunogens in organ xenotransplantation. In total, we selected 101 candidate genes, which we suggest may be important in the antibody-mediated response to the pig microvasculature (Fig. 3E).

### Silencing porcine candidate genes is effective in reducing antibody binding to DKO RMEC

**Figure 4:**
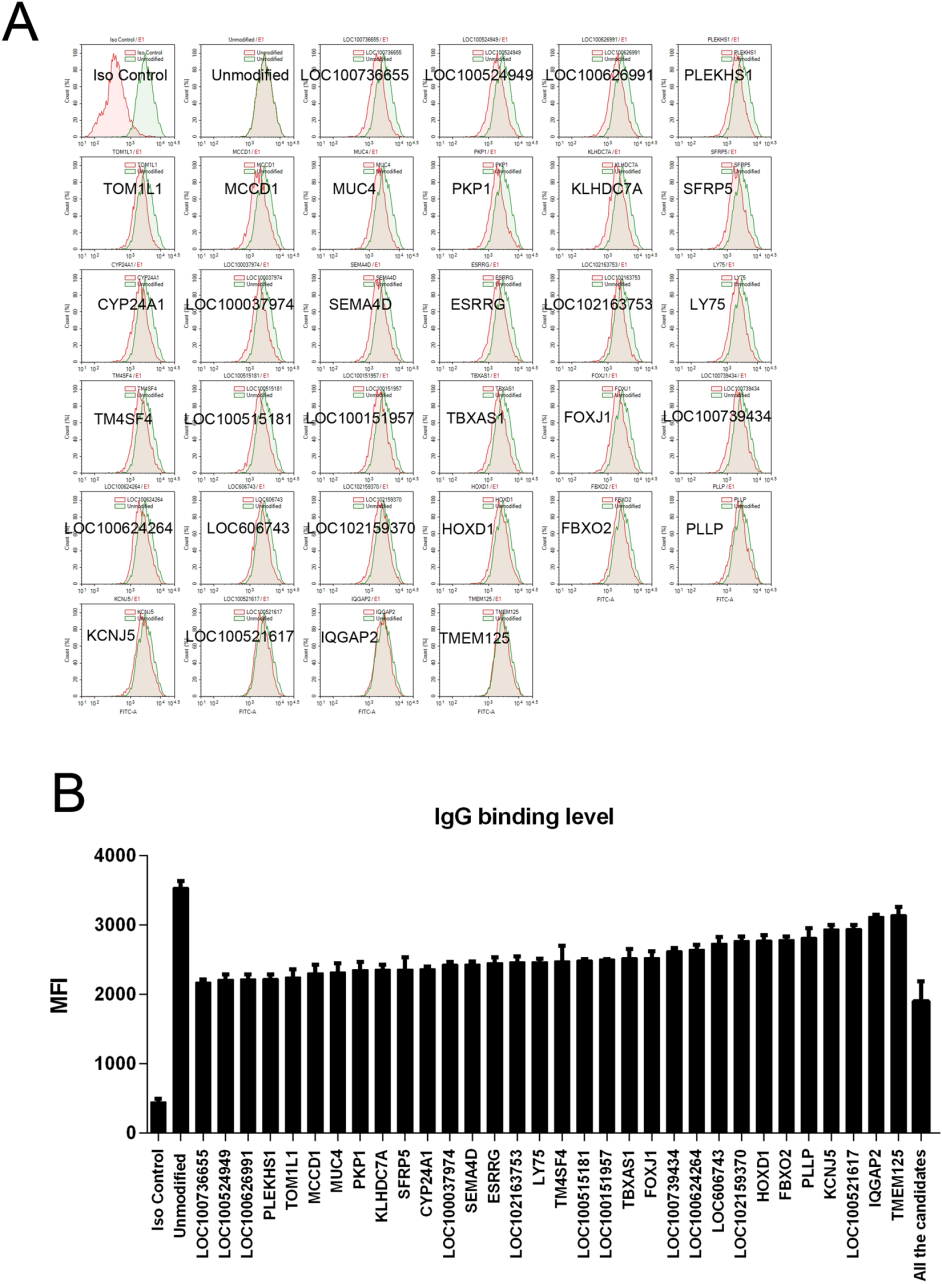
Candidate genes that, when individually deleted, led to a reduction in serum antibody binding to RMEC The 101 candidate genes were individually knocked out by CRISPR-Cas9 technology in pig RMEC. (**A**) Monkey serum antibody binding was determined by incubating the RMEC with monkey serum drawn 8 weeks after immunization. The negative control (Iso control) is RMEC incubated with non-immunized serum. The positive control is unmodified GGTA1/CMAH DKO RMEC incubated with immunized serum from the 8th week. The remaining figures document the reduction in antibody binding following knockout of a single individual candidate gene. (**B**) Data from (A), i.e., IgG binding (MFI) to RMEC, displayed together.

The 101 candidate genes in the primary RMEC were knocked-out by CRISPR-Cas9 technology individually, followed by measurement of antibody binding to the individual RMEC, using serum drawn at week 8 after immunization of the monkeys. After knockout of a single gene, serum antibody binding was significantly reduced in 32 cases (Fig. 4A,B). The information of the candidate genes is listed in Table 1. When all 32 genes were knocked out, antibody binding was significantly reduced (Fig. 4B).

**Table 1:** Information on 32 candidate genes

Gene name	IgG binding value 222vV(MFI)	Gene_ID	Gene description
LOC100736655	2178 ± 72	100736655	uncharacterized
LOC100524949	2219 ± 124	100524949	E74 like ETS transcription factor 3
LOC100626991	2225 ± 108	100626991	MACC1, MET transcriptional regulator
PLEKHS1	2228 ± 110	100157918	pleckstrin homology domain containing S1
TOM1L1	2248 ± 205	100522701	target of myb1 like 1 membrane trafficking protein
MCCD1	2312 ± 205	100157598	mitochondrial coiled-coil domain 1
MUC4	2323 ± 221	100157344	mucin 4, cell surface associated
PKP1	2360 ± 190	100623590	plakophilin 1
KLHDC7A	2362 ± 112	100622416	kelch domain containing 7A
SFRP5	2366 ± 291	100153176	secreted frizzled related protein 5
CYP24A1	2370 ± 52	397145	cytochrome P450, family 24, subfamily A, polypeptide 1
LOC100037974	2436 ± 59	100037974	cartilage acidic protein 1
SEMA4D	2437 ± 72	100152598	semaphorin 4D
ESRRG	2458 ± 142	100622056	uncharacterized
LOC102163753	2467 ± 150	102163753	uncharacterized
LY75	2472 ± 73	595126	lymphocyte antigen 75
TM4SF4	2486 ± 371	100522400	transmembrane 4 L six family member 4
LOC100515181	2495 ± 26	100515181	uncharacterized
LOC100151957	2508 ± 7	100151957	par-6 family cell polarity regulator beta
TBXAS1	2527 ± 227	397112	thromboxane A synthase 1
FOXJ1	2533 ± 152	100623071	forkhead box J1
LOC100739434	2628 ± 76	100739434	uncharacterized
LOC100624264	2649 ± 121	100624264	laminin subunit gamma-2-like
LOC606743	2740 ± 152	606743	adenosine A1 receptor
LOC102159370	2775 ± 105	102159370	laminin subunit alpha-1-like
HOXD1	2782 ± 127	100157662	homeobox D1
FBXO2	2787 ± 91	100511640	uncharacterized
PLLP	2822 ± 230	100516614	plasmolipin
KCNJ5	2943 ± 101	397448	potassium voltage-gated channel subfamily J member 5
LOC100521617	2948 ± 95	100521617	myelin and lymphocyte protein
IQGAP2	3125 ± 40	100519536	IQ motif containing GTPase activating protein 2

## Discussion

The humoral immune system provides a significant barrier to solid organ transplantation as a result of antibody-mediated recognition of non-self proteins and carbohydrates expressed on the vasculature of the pig^25, 26^. Research over the last few decades has established that vascular endothelial cells are a primary target for xenograft recipient immune responses^27^. There is also increasing recognition that a functional microvasculature is an important determinant of the long-term health of transplanted solid organs^28^.

Hyperacute rejection, which is induced by anti-Gal antibodies, has been overcome by eliminating expression of Gal antigens in the pig^8, 29^. Two other carbohydrate xenoantigens, N-glycolylneuraminic acid (Neu5Gc, the product of CMAH) and Sda (the product of β1,4 N-acetylgalactosaminyl transferase 2), have been identified^30^. Neu5Gc is an important pig antigen to humans.

The main purpose of the present study was to try to identify potential antigens that might be associated with the development of DXR in the absence of expression of Gal. We generated DKO pigs deficient in Gal and Neu5Gc. Human antibody binding and complement-dependent cytotoxicity are both significantly reduced to these cells^23^. Nevertheless, DXR can be induced in the presence of nonGal endothelial cell membrane antigens, and/or may also result in chronic rejection and/or thrombotic microangiopathy, and/or graft loss. We established a pig endothelial cell immunization model in monkeys which reflects pig kidney xenotransplantation in that the monkey was sensitized to both RMEC and AEC as it would after a kidney transplant. As no immunosuppressive therapy was administered to the monkeys, this model allowed investigation of the immune response^31^. We suggest that the model may be helpful in identifying candidate antigens that may be associated with the development of DXR and/or thrombotic microangiopathy.

Serum antibody binding to RMEC proved to be significantly higher than to AEC, suggesting that the immunogenicity of RMEC is greater than of AEC. We suggest that this is an important observation as most groups use pig AECs in *in vitro* assays when determining the primate antibody response to pig cells. Our study suggests that assays based on pig AECs may significantly underestimate antibody binding to a pig kidney *in vivo*. The increased antibody binding to pig RMEC suggests that there is greater injury to these cells *in vivo*, leading to the development of fibrin-platelet thrombi, resulting in ischemic injury to the graft. It may be of relevance that Knosalla *et al*. noted significant differences in gene expression between kidney and heart, and this may account for the more rapid development of thrombotic microangiopathy and consumptive coagulopathy reported after pig kidney transplantation than after heart transplantation^32^. RMEC may be more immunogenic than the vascular endothelial cells of the coronary system (which may be more like AEC).

The progression of thrombotic microangiopathy appears to correlate with an increase in immunoglobulins and complement deposition in the graft^33^. Chen *et al*. were the first to demonstrate that baboon recipients of GGTA1-KO porcine kidneys treated with inadequate conventional immunosuppressive therapy rejected their xenografts relatively rapidly (by day 16) in the presence of high titer elicited cytotoxic antibodies directed to nonGal epitopes. They hypothesized a harmful role for anti-nonGal antibodies when GGTA1-KO organs were transplanted, although the specificity of such antibodies was not defined^34^.

In our study, we established a new system of identifying antigens based on the antibody response to immunization with RMEC and AEC, combined with RNA-seq to focus on both species- and tissue-specificity genes. We anticipated that the antibody binding level would be reduced if one or more antigens was knocked out. Thirty-two genes were individually knocked out, and the antibody binding to the modified cells was assessed. In all cases, antibody binding was reduced (in some cases by > 40%), confirming the relative immunogenicity of the identified gene products.

The selected 32 candidate genes are not found in the human genome, so they may be important antigens when pig organs are transplanted into humans. To produce a pig without any of the selected genes is not technically possible currently. In the future, we will identify the most important antigens in regard to the immune response. As few or none of the antigens may be of great importance in this respect, to prevent sensitization, immunosuppressive therapy may be the most effective strategy. Novel immunosuppressants are needed, for example, anti-CD40 monoclonal antibody. However, the present study provides novel antigen information that may be of value in indicating genes to be deleted in the future.

Confirmation of identification of these porcine gene products may create new opportunities for genetic modification of the organ-source pig to reduce the level of xenograft antigenicity and enhance resistance to DXR^35^, resulting in long-term xenograft survival. Further studies will be carried out to determine the most important antigens in stimulating an immune response. They could then either be knocked out or the response to them may be prevented by adequate immunosuppressive therapy.

## Electronic supplementary material

Supplementary Information
