# Supplementary material for: Potential Antigens Involved in Delayed Xenograft Rejection in a Ggta1/Cmah Dko Pig-to-Monkey Model
Source: Sci Rep. 2017 Aug 30;7:10024. doi: 10.1038/s41598-017-10805-0 (PMC5577312; doi:10.1038/s41598-017-10805-0)
Supplement: Supplementary file 1 — Supplementary Information [file 41598_2017_10805_MOESM1_ESM.pdf]

POTENTIAL ANTIGENS INVOLVED IN DELAYED XENOGRAFT REJECTION IN A GGTA1/CMAH DKO PIG-TO-MONKEY MODEL

Junfang Zhang[1,2], Chongwei Xie [1,2], Ying Lu[1,2], Ming Zhou[1,2], Zepeng Qu[1,2], Da Yao[1], Chuanghua Qiu[1], Jia Xu[1], Dengke Pan[3], Yifan Dai[4],Hidetaka Hara[5], David K.C. Cooper[5], Shanshan Ma[2], Mingtao Li[2], Zhiming Cai[1], Lisha Mou[1]

1. Shenzhen Xenotransplantation Medical Engineering Research and Development Center, Institute of Translational Medicine, Shenzhen Second People's Hospital, School of medicine, Shenzhen University, Shenzhen, Guangdong, 518060, China

2. Department of Biochemistry, Zhongshan School of Medicine, Sun Yat-sen University, Guangzhou 510080, China

3. Key Laboratory of Farm Animal Genetic Resource and Germplasm Innovation of Ministry of Agriculture, Institute of Animal Science, Chinese Academy of Agricultural Sciences, Beijing 100193, China

4. Jiangsu Key Laboratory of Xenotransplantation, Nanjing Medical University, Nanjing 210029, China

5. Xenotransplantation Program/Department of Surgery, The University of Alabama at Birmingham, Birmingham, AL 35233, USA

Figure S

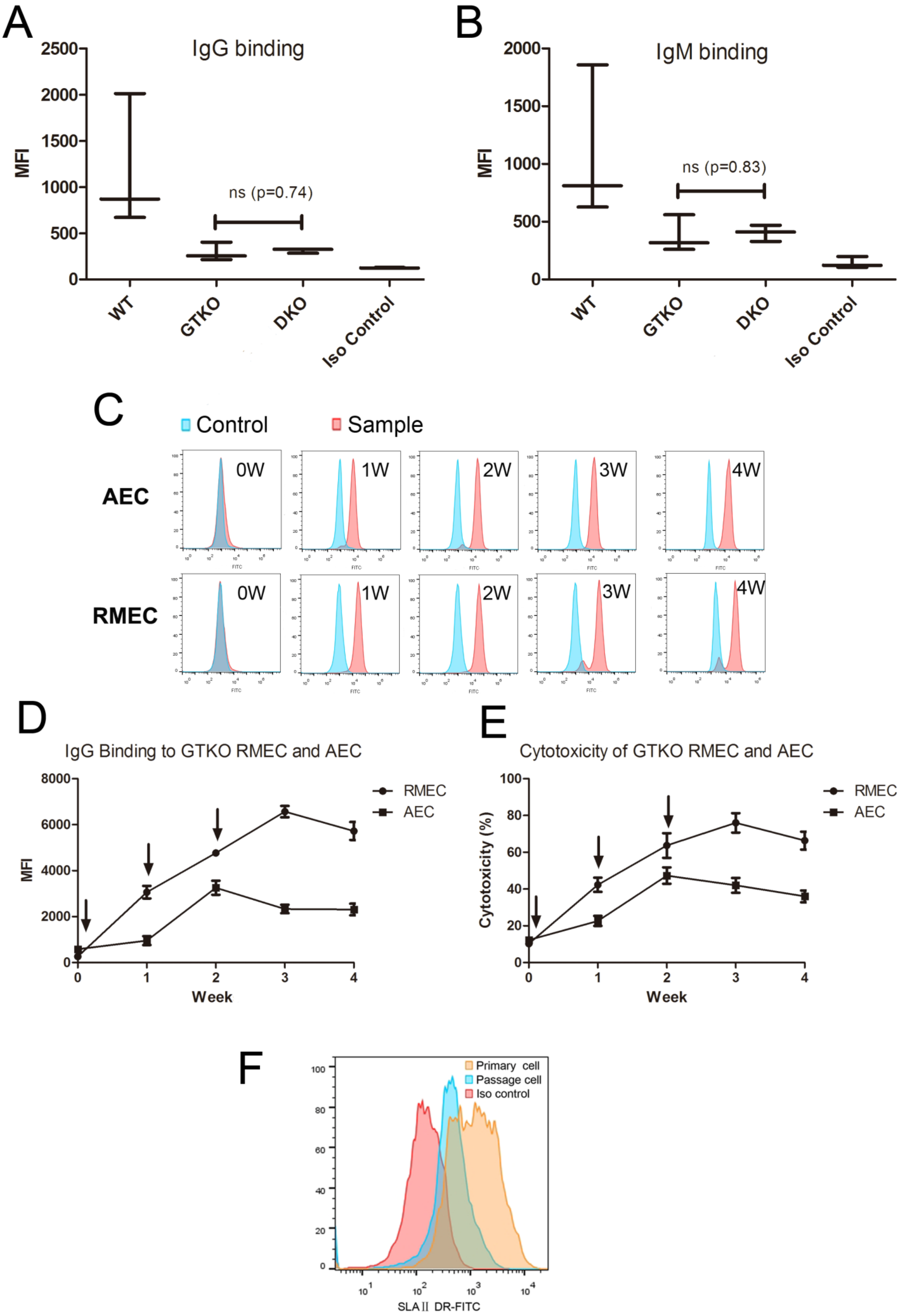

A
IgG binding
MFI
ns (p=0.74)
WT
GTKO
DKO
Iso Control
B
IgM binding
MFI
ns (p=0.83)
WT
GTKO
DKO
Iso Control
C
Control
Sample
AEC
RMEC
0W
1W
2W
3W
4W
D
IgG Binding to GTKO RMEC and AEC
MFI
Week
RMEC
AEC
E
Cytotoxicity of GTKO RMEC and AEC
Cytotoxicity (%)
Week
RMEC
AEC
F
Primary cell
Passage cell
Iso control
SLA II DR-FITC

Figures Legends

Supplementary Figure

(A) IgG binding of non-sensitized monkey serum to WT, GGTA1-KO, DKO RMEC.

(B) IgM binding of non-sensitized monkey serum to WT, GGTA1-KO, DKO RMEC.

(C) We sensitized a monkey with GGTA1-KO RMEC and AEC (weekly x3), and collected serum for IgG binding by flow cytometry. (‘Controls’ are isotype controls)

(D) Statistical analysis of IgG binding to GGTA1-KO RMEC and AEC using sensitized monkey serum. Binding to RMEC was significantly higher than to AEC ($p<0.05$).

(E) Percentage cell death (% cytotoxicity) of GGTA1-KO RMEC and AEC. Cell death of RMEC was significantly greater than of AEC ($p<0.05$).

(F) Expression of SLA class II on primary cells and after passage.

Supplementary Table S1: Information on guide-RNAs

Number	Gene Name	gRNA1	gRNA2	gRNA3
1	SLC1A6	GATTCCCTTGTCTTATTCGA	AGAACTGGCGAACACAACAC	ATCAGCCGCCTTCCTAGCAA
2	LOC100521617	CTGACCGCTCGCTTGCTCGG	GGCCAGGGAGCCACCGCGCG	GCTTGCTCGGCGGAGCCCAG
3	LAMB3	CGGAGGAGCGCTCGATCAGC	CCAGGTCTTGCCGAAGTCGG	CCGCCAGGTCTTGCCGAAGT
4	LOC100626947	CCAGGGCTGGCCATGCAGGC	GAGCCCAGGGCTGGCCATGC	GAGTGTGCGCAGGGGTGGCA
5	LOC100736655	GCGGAGCTGAAGGACCGGTT	AATACGCGGAGCTGAAGGAC	CCGCCTCCATCTTGACGCTC
6	SEMA4D	TGGTCGGGAAATTCCAGGTG	ATGCAGCTGGTCTGGGACGG	CGACTGGGATGCAGCTGGTC
7	LOC100524949	CGTCCATGTCACAGCGCGAG	GTCACAGCGCGAGAGGTCGA	GCGCGAGAGGTCGATGGTGC
8	HPN	TAGGCCTGGTTGCCTACCTG	GGCGTGGGGCCTGCTAGGCC	GCGGCGGCGTGGGGCCTGCT
9	IQGAP2	AAAAGCTCGTTCTGATACAT	CTGATACATTGGAGCAGCAA	GGGCAAGCAGCGTGTTCTCA
10	SLC2A2	CACCAATGTCGTATCCAAAC	AATGTCGTATCCAAACTGGA	AGGAACCCAGCACGGCAGTG
11	LOC100037974	GAACATGGGTTCAGCCCGCT	TGAACATGGGTTCAGCCCGC	TTGGTGACTGCCGTGAACAT
12	LOC100626812	TGGACTTGCGCATGGCGTAC	CAGAGGCCAGGTCGTAGCGG	GCGGTGGATGGACTTGCGCA
13	SFRP5	TCGTACTCCTCGCCGTGTGC	TTGGGCGACCTCAGCGGCCG	TCAGCGGCCGGGGAGCGTCG
14	LOC100737124	GGCCATGGACGGAAGGTGCG	GGCCCCCGGCCATGGACGGA	TGCTGGCCCCCGGCCATGGA
15	PLLP	GCAACTCGCGGCGTCGGGGG	TCGCGGCGTCGGGGGAGGCT	CTCGCGGCGTCGGGGGAGGC
16	LOC100151957	CGCTGCCCGCTCCGTGCCGG	AGCCGCTGCCCGCTCCGTGC	CCGCTCCGTGCCGGTGGCTG
17	LOC100737810	CTTCTTGTATTGGTCCTAAG	GCCGTGATACTTCCTCTCTA	TTCTTGTATTGGTCCTAAGT
18	LOC100624264	GAACCTGTAACCCCAAGGTA	TGTAACCCCAAGGTAGGGAA	AGAACCTGTAACCCCAAGGT
19	CDH15	GCAGTCCATGAAAACTACTG	CAGTCCATGAAAACTACTGT	AGTCCATGAAAACTACTGTG
20	MCCD1	AGACGCAAGACGTGGTCAGC	CGAGAAAAAGACGCAAGACG	CACGTCTTGCGTCTTTTTCT
21	LOC102159370	AGGTATCACGTCGGAACTGA	AAAGCATGGAGGTATCACGT	AGAATGCTAATCAAATTACG
22	MUC4	TAAGCAGCTCAGAAACACCC	TGAGGGGGGTCCACCAGAGG	CCACCAGAGGAGGGTCCCCT
23	KIAA1211L	AGCAGCGGTGTTTCGCGATC	CTTAATGTCCATCACCCTTG	GCCATCCTCTCCGAGGCCTT
24	LOC102164483	GGAGGACAAGGTTGTTGATA	CAAGGTTGTTGATATGGTCA	AAGGTTGTTGATATGGTCAA
25	LOC102165085	ATCATCGACATGACAGCGGG	GGCAGATAAATTCCGACCAG	TCATCGACATGACAGCGGGA
26	TMEM125	TGCTCGGCCAGCACATCCGG	TGCCGAGCGCCGCGGCTGCT	AAGCAGAGTGCCGAGCGCCG
27	LOC102157775	CACGGTGAACGTGTAGCTCT	GCAGAGAGACTGCGGCACGG	CCTCTCTCCAAGTGCCGCCG
28	PPARGC-1	TAACTTAGCTGAGTGTTGGC	TGAGGCGGAGGGGTGCCGTC	TCTACTTAAGAAGCTCTTAC
29	TOM1L1	TGGCCCACGGAGGTCGCGTA	GGCCCACGGAGGTCGCGTAC	GACCCGTACGCGACCTCCGT
30	FRMD4B	TCATCTCTGAACCATGATCT	CATGGTTCAGAGATGACAGA	GTTCAGAGATGACAGAAGGC
31	PKP1	CTGGAAGCATTCGTAGGCCA	GCGCTGCCGGCCCGACGTAC	ACGTACCGGTCTTCATCTTC
32	CKMT1B	TGGCGGCTCGAATAGGTTCC	CGTTCACTGGCGGCTCGAAT	CTGGCGGCTCGAATAGGTTC
33	DOCK8	TGATCTTGAGCGCGAACGCG	CGTTCGCGCTCAAGATCAAC	TTATGCTGTGTCGATGGTAC
34	FBXO2	GACGGTGACCCAGAGAGCGT	TCTGGCTGGCCCACGCTCTC	CTGGCTGGCCCACGCTCTCT
35	ELMOD1	CTTTCTGAGAATGTTGATCC	AGTAGAAATACAGGCACACC	CTGGAAGATGTGAACTGCAA
36	PLEKHS1	CCCAAACCTCAGAAGACTCC	TAAAATGTAAATTGTCTGCC	CCTGGAGTCTTCTGAGGTTT
37	DACH1	GTCGGAGGGATCAAAGCCGC	ACGTGGAGATCGGGGGTTGA	GAAGCAGACGTGGAGATCGG
38	KLHDC8A	GTGGCGCCAGGCGCTTCCAC	CACTGGAAGTCCTTGACGTT	GAGCAGTAGACCCGGCGGCT
39	EPB41L5	ACGCATCGACCTCCGACCGA	GACTTCGCATCTCCAGCCGC	TACGCATCGACCTCCGACCG
40	TJP3	GCGGGGGTCCTTGCTGATCG	GCAATGCCAAAGCCCCGGCG	TTGCAATGCCAAAGCCCCGG
41	FAM20A	CAGGCGGTCCCGGCGCAGTC	AGGCGGTCCCGGCGCAGTCC	GCAGGGTCAGCAGGCGGTCC
42	LOC100515181	TTCCGGTTGGATCCGGCAAG	GAGAATATTCCGGTTGGATC	TCCAGTGAGAATATTCCGGT
43	LOC100620451	GTTGTGGGATAATTTGGCGA	CCAAAAAAGAGTTCGCGGCG	TTGTGGGATAATTTGGCGAA
44	LOC100737363	CCCCACCAGCAGTAGGAACT	CCCCCACCAGCAGTAGGAAC	CCGGAACCCCCACCAGCAGT
45	LOC606743	CCACCGTCTTGTACCTAGTG	GTCTTGTACCTAGTGTGGTC	TCTTGTACCTAGTGTGGTCA
46	LOC100514841	CGCTGGCGCCCGGCTTTGTT	GCTGGCGCCCGGCTTTGTTT	CTGGCGCCCGGCTTTGTTTG
47	CYP24A1	CGAAGTGGAGGCGTCTAAGA	GGAGGCGTCTAAGAGGGTAG	AATCTGGAGCCGCGAAGTGG
48	LOC100739411	TCATAGTAAGGCCCGACACC	TATCCGTGGTTTTCATAGTA	CACTTTCAGGTTGGTATCCG
49	ZNF385B	AGAGGAATTGTCAGGGCTTA	CATCTTCAGAGGAATTGTCA	CCATCTTCAGAGGAATTGTC
50	LOC100627251	ATCAGAGGAATCGCAATCCA	CAGACATTTCTACAAGTGAT	TCAGACATTTCTACAAGTGA
51	LOC102168166	AGGGCTTGCCCTTAACGTCG	CTTGTAGTTGCGGCTGATCA	TTGTAGTTGCGGCTGATCAA
52	LRCH2	TGACCGAAAAGAGTCGGTAC	AAAAGAGTCGGTACCGGGAT	AAAAGAGTCGGTACCGGGAT
53	LOC100739434	CAGATATCATCCATCATATC	ATTTCTAGTACCAGATATGA	GGTCCAACACCCCGGCCCCT

Number	Gene Name	gRNA1	gRNA2	gRNA3
54	CLGN	CCACGGCTTTCGGCTATGTT	CCAAACATAGCCGAAAGCCG	CACGGCTTTCGGCTATGTTT
55	LOC100623659	CAGGAACTCGGCTGTCTCCG	TGCGTCCCAAGCTGCCCGTC	GCTGCATCGCATGCGGCAGC
56	LOC100518964	CTCTAAGTAGCCTGAGCCGC	GAGCAGTCCCGACTCGGTTC	CCACCTGAGCAGTCCCGACT
57	LOC100525734	AGGGCTTGCCCTTAACGTCG	CTTGTAGTTGCGGCTGATCA	GAAGTGCTCGATCTCGCTCA
58	LOC100624418	AACAGGAGCCGCTCGTTTGG	CTCGTTTGGGGGCGCGTCCA	GAACAGGAGCCGCTCGTTTG
59	ESRRG	GTCAATGTGTCGGTCTTTGT	CTTGATGAAGGACGAACAGC	AACAGCTGGAGTCAATGTGT
60	LOC100521253	CGTCCACGATAGAGCCATAG	GTCCACGATAGAGCCATAGC	TCCACGATAGAGCCATAGCG
61	LOC100523750	CGAGGCGCTGGGCCGCTACG	TGCGCGCCTCCTCGGCGTCG	CACCGTACGCGCCAGCGGCC
62	ENPP5	AGCAGAACTTTTTGCTGGTC	TTCATTTGATGGATTCCGTT	TTTCATTTGATGGATTCCGT
63	LOC100622420	CTGAGCTCTTGCCGGCGCTC	TCCTCTGGTGAACCGTCTGC	TCCTCTGGTGAACCGTCTGC
64	LOC100151826	GTCCCAGGTCACCCGGTGGT	GAACACCTGCGCCGAGTCCC	GAGCAGGACTCCATCGCGGA
65	LOC100628231	TGTAGCTTACTATTCCAAGC	CCATTCTTCTGACAGAGGTA	ATCCTACAGAAAATCCAACC
66	LOC100737399	GGAACTTGACAACCTTCCCG	AACTTGACAACCTTCCCGGG	GAACTTGACAACCTTCCCGG
67	LOC102161581	TTGTGGGCTGCTCGCTTGGC	GCTGCATCGCATGCGGCAGC	GACATTGTGGGCTGCTCGCT
68	PTPN6	TCGCCCTGGTTCTTGCGACT	GTCGCCCTGGTTCTTGCGAC	GTCTCGGTGAAACCACCCAC
69	LOC100737014	GCATGGTGGCCCCGCAGCGG	GGCGTGGGGCCTGCTAGGCC	GGAGCATGGTGGCCCCGCAG
70	TBXAS1	CCTCTGAGTGGGTACTATCT	CCAAGATAGTACCCACTCAG	GTGGGTACTATCTTGGTCGC
71	IGSF5	CTTCACCACCGTGACGTTCC	TTCACCACCGTGACGTTCCT	GCTGGCTGCGCTGGTCGTTC
72	ATP6V1B1	AGCATACTGGGCAAACTGGC	GTTAACAATCTCAGCATACT	AGTTAACAATCTCAGCATAC
73	HTR1D	TCCTCTATGGCCGGATCTAC	CCCTGTAGATCCGGCCATAG	CGCTTCCCGTAGAGTGACGG
74	HOXD1	GCGTCGGCGCGGCAGAACTT	GGCCGCACGACATGTAGTCC	GGCGTCGGCGCGGCAGAACT
75	SLC12A1	TGAAAGCGATTGGTATTACC	GACATTGACTTGAAAGCGAT	TGAAAGCGATTGGTATTACC
76	EMID1	GGCTCCCCCGACGCTCCACG	GCACATGGCACGAGATGGTG	GTTCTGCACATGGCACGAGA
77	TMEM176B	CGCGCACCGTCAACGTGTCC	GGTGGATGTGGATGTCGATG	GCAGTGCCAAGGCGGATTCC
78	LOC100524999	GTGCCCCCGGCGCCGTATCT	TATCTTGGCTGCGAAACAAC	GTCACTAACCGCTGTGCCCC
79	B4GALNT2	CTGTATCGAGGAACACGCTT	ACATAAAGAGTCCAACGCTC	TGAGGATCGACAGACATCTA
80	CAPN6	TGAATCGTTCTCAGGCAAAA	TAGAAAAGTGAATCGTTCTC	TCGTTCTCAGGCAAAAAGGT
81	KLHDC7A	GCCTGTAGGCTGCGGTCACC	CTTGTACAGCCTGTAGGCTG	CCTCGACTTGTACAGCCTGT
82	LOC100511825	TGGCGGCACGATGATGATTT	CTATCCCAACAGCACTCCAC	CTCCACAGGAGCATGAAATA
83	FAM169A	GGCATTCCCTGTGGATATGC	TATGCTGGATAATTGCAGTC	CAATTATCCAGCATATCCAC
84	LOC100738758	ACAGAAAGGCAACGTTGGCT	CTGTAGCTGATGGCCTGTAA	GGCTTGGTGACTGTAGCTGA
85	TM4SF4	GGGGAATGAGGGTGCCCCCC	GAGGGTGCCCCCCAGGCACT	AAAATAGCAGGATGTTAGCC
86	LOC100626991	CTAATGCTACAAATCCATTC	GTCTGTTTCTAACCCATTTT	TAAAGGATCTTCCTTCAAGA
87	LOC100623790	GGAGGACAAGGTTGTTGATA	CAAGGTTGTTGATATGGTCA	AAGGTTGTTGATATGGTCAA
88	RAVER2	CCGGACGTCGTCGCGCGCCT	GACGTCGTCGCGCGCCTCGG	TCGTCGCGCGCCTCGGCGGA
89	ASXL3	TGGGGTGTTTTTCTAGTGCC	GGAAGAAGAAGGACCGCACC	CTTTGCTGTCATTGGTGAGT
90	LOC100514082	CAGCGCCGTCGCCGCCCCTG	GGCTGTCGCCGCCAGCTCCC	GAGCTGGCGGCGACAGCCAC
91	LOC100520836	ACGGCCAAGTGGCGTCTGCG	CGGCCAAGTGGCGTCTGCGG	CACGGCCAAGTGGCGTCTGC
92	LOC100738350	GCTTCACCGTCATACTCTTC	GCTCCAGCGGGACACCCTTC	ACGCGGGCGCGGTGCTCCAG
93	RGS14	TTGGGGACGCCCAGGTGCTT	TGCGTCCGTTGGGGACGCCC	GTTGGGGACGCCCAGGTGCT
94	LOC100153981	TTCTGTGGGGCCGAGATCTC	CACGGTGCTGGAAGTTCTGT	TCTGTGGGGCCGAGATCTCA
95	LY75	GCAGCTCCGCCACACGGCGA	CAGCTCCGCCACACGGCGAG	AGCAGCTCCGCCACACGGCG
96	C8H4orf19	CCAGGGGAGGGCACTTGAAC	TTCGTTGACATAACCAGGGG	TCGTTGACATAACCAGGGGA
97	FOXJ1	CTCTAGGCCGCCCTCCGGCC	GGCTCCTCTAGGCCGCCCTC	GCTGGTCAGGCTGTCATCCA
98	KIAA1147	CCAGCGGCGGGGAGACGGCG	GCCTCCAGCCTGCGGGTCCG	CGGCCTCCAGCCTGCGGGTC
99	SESN3	GGTAGTTGGCGGCGGCCGAC	AGGTAGTTGGCGGCGGCCGA	GTTGGTACAGAGCAGGTAGT
100	KCNJ5	GTCCTGGTTCATAGCATTCC	TGACCCCAATCTCCATGTCC	GGGAATCTTCTTAGGGTCCC
101	CPVL	ATCAAGAGGGCCAGCAAAAC	TACCAGGGCTGAGCATCAAG	GAAACAGGCCATCACTACCA

Supplementary Table S2: Genes expressed in DKO RMEC, but not in GTKO RMEC

Gene_ID	Gene Name	Value (DKO-RMEC)	Value (GTKO-RMEC)	p_value
ENSSSCG00000001239	ZNRD1	14.9565	0	0.00005
ENSSSCG00000013902	-	4.92228	0	0.00005
ENSSSCG00000015142	-	2.13301	0	0.00005
ENSSSCG00000021650	-	14.0226	0	0.00005
ENSSSCG00000023256	-	48.5251	0	0.00005
ENSSSCG00000029400	RBP5	9.62054	0	0.00005
ENSSSCG00000029556	MAP3K4	3.97507	0	0.00005
ENSSSCG00000030938	CH242-38B5.3	11.5388	0	0.00005
ENSSSCG00000005167	SLC24A2	1.67768	0	0.00015
ENSSSCG00000012132	ASB9	5.34358	0	0.00025
ENSSSCG00000017959	-	1.50703	0	0.00135
ENSSSCG00000000997	PPP1R3G	1.73303	0	0.0014
ENSSSCG00000001711	CLIC5	0.667775	0	0.0018
ENSSSCG00000008627	-	0.778969	0	0.0019
ENSSSCG00000010577	ELOVL3	1.31878	0	0.00285
ENSSSCG00000015068	APOA4	0.71314	0	0.0033
ENSSSCG00000016296	-	2.45053	0	0.0033
ENSSSCG00000026706	-	0.697953	0	0.0033
ENSSSCG00000000468	-	5.60476	0	0.00385
ENSSSCG00000004236	-	1.29338	0	0.00385
ENSSSCG00000030876	RBP5	1114.87	0	0.00385
ENSSSCG00000006141	CA3	0.756662	0	0.0039
ENSSSCG00000008626	-	0.941947	0	0.00465
ENSSSCG00000008084	-	4.64556	0	0.00545
ENSSSCG00000027191	-	9.00664	0	0.00545
ENSSSCG00000002453	-	0.679599	0	0.0067
ENSSSCG00000025483	-	0.749387	0	0.00715
ENSSSCG00000028770	RCVRN	1.08145	0	0.00715
ENSSSCG00000001096	C6orf229	1.26974	0	0.0087
ENSSSCG00000011339	CSPG5	4.07126	0	0.0087
ENSSSCG00000011808	SST	1.56473	0	0.0087
ENSSSCG00000021215	-	715.382	0	0.0087
ENSSSCG00000003688	-	10.4904	0	0.0115
ENSSSCG00000013153	-	0.736059	0	0.0115
ENSSSCG00000015125	-	0.640763	0	0.0115
ENSSSCG00000023849	CTRL	0.97851	0	0.0115
ENSSSCG00000028292	-	1.66045	0	0.01895

Supplementary Table S3: Genes expressed in DKO AEC, but not in GTKO AEC

Gene_ID	Gene Name	Value (DKO-AEC)	Value (GTKO-AEC)	p_value
ENSSSCG00000005979	ANXA13	4.33028	0	5.00E-05
ENSSSCG00000010184	AGT	3.43816	0	5.00E-05
ENSSSCG00000010411	RASSF4	1.83245	0	5.00E-05
ENSSSCG00000010966	CCL19	19.2357	0	5.00E-05
ENSSSCG00000012179	ZFY	2.80177	0	5.00E-05
ENSSSCG00000017448	KRT19	5.65056	0	5.00E-05
ENSSSCG00000016473	EPHB6	1.17642	0	0.00025
ENSSSCG00000007100	SLC24A3	0.932555	0	0.0005
ENSSSCG00000002835	TOX3	1.40823	0	0.0007
ENSSSCG00000006503	GPCR142	2.8158	0	0.00095
ENSSSCG00000016868	ANXA2R	4.52152	0	0.001
ENSSSCG00000007405	WFDC2	5.53122	0	0.00115
ENSSSCG00000009738	GALNT9	0.729682	0	0.0013
ENSSSCG00000008141	ST6GAL2	1.19954	0	0.0016
ENSSSCG00000015901	GRB14	1.28692	0	0.00175
ENSSSCG00000016129	GPR1	1.9809	0	0.00235
ENSSSCG00000016841	SLC1A3	0.771151	0	0.00275
ENSSSCG00000003287	BRSK1	1.49936	0	0.00285
ENSSSCG00000008962	EREG	5.31068	0	0.00305
ENSSSCG00000008965	BTC	3.00574	0	0.00305
ENSSSCG00000013461	C19orf35	0.823242	0	0.00305
ENSSSCG00000011148	-	1.36261	0	0.0038
ENSSSCG00000017982	HES7	1.50567	0	0.0038
ENSSSCG00000009801	BCL7A	1.0553	0	0.00405
ENSSSCG00000020838	-	0.587483	0	0.00405
ENSSSCG00000007678	COL26A1	0.69509	0	0.0044
ENSSSCG00000000857	IGFI	0.813	0	0.0064
ENSSSCG00000007978	HBA	2.06713	0	0.00725
ENSSSCG00000016627	-	0.856845	0	0.04075
ENSSSCG00000016903	GZMA	1.02852	0	0.04075
ENSSSCG00000020786	GNG7	132.928	0	0.04075
ENSSSCG00000007730	-	1.31339	0	0.0423
ENSSSCG00000002536	-	1.02151	0	0.04595
ENSSSCG00000007774	CTF1	1.1562	0	0.0462
ENSSSCG00000005474	SAL1	0.623272	0	0.0483
ENSSSCG00000010338	DYDC1	1.18176	0	0.0483
ENSSSCG00000014229	PRR16	1.62931	0	0.0483
ENSSSCG00000016999	TLX3	0.615698	0	0.0483
